# Motorcyclist risky riding behaviors and its predictors in an Iranian population

**DOI:** 10.5249/jivr.vo112i2.161

**Published:** 2020-07

**Authors:** Kamal Hassanzadeh, Shaker Salarilak, Homayoun Sadeghi-Bazargani, Mina Golestani

**Affiliations:** ^*a*^ Department of Epidemiology, School of Health, Tabriz University of Medical Sciences, Tabriz, Iran.; ^*b*^ Department of Public Health, Islamic Azad University of Tabriz, Medical school, Tabriz, Iran.; ^*c*^ Research Center for Evidence-Based Medicine, Tabriz University of Medical Sciences, Tabriz, Iran.; ^*d*^ Road Traffic Injury Research Center, Tabriz University of Medical Sciences, Tabriz, Iran.

**Keywords:** Motorcycle riders, Risky behavior, Speed, Iran

## Abstract

**Background:**

Motorcyclist’s behavior plays an important role in increasing the mortality rate caused by traffic crash. Identifying the risky behaviors of motorcycle riders is essential to maintain and improve the health of motorcycle riders and other community members. The aim of this study was to determine the riding patterns and risky riding behaviors of motorcycle riders in Bukan as a marginal small-sized Kurdish populated district in North-West of Iran and investigating some predictors of it.

**Methods:**

In this cross-sectional, 340 motorcycle riders of Bukan were studied. By referring to city health center and preparing the city map, the entire city was divided into 14 clusters based on the areas covered by the health centers. Then, 7 clusters were randomly selected out of these 14 clusters. Motorcycle riding behavior questionnaire (MRBQ) was used to study the risky behaviors of motorcycle riders while riding. Both bivariate and multivariate regression analysis methods were used to study the associations.

**Results:**

All participants were male. Their mean age was 30.2 (SD=9.1). The most common risky behaviors possessed by at least 23% of motorcycle riders included 1) inappropriate control of motorcycle when turning, 2) taking another person without helmet by motorcycle, 3) riding without helmet, 4) taking more than one person by motorcycle, 5) exceeding the permissible speed outer city, 6) exceeding the permissible speed inside the city and 7) carrying heavy load by motorcycle. Mean normalized MRBQ score was 30.5 (SD=11.2). Based on multivariate analysis, age, lacking a riding license, riding experience and average amount of riding were the independent predictors of risky riding score.

**Conclusions:**

Among the predictive factors that led to high risk behaviors in the studied motorcyclists were low age, marital status, low driving experience, low education, non-use of safety equipment lack of certification. This can be done by increasing drivers' awareness of laws and regulations and promoting the culture of traffic safety to prevent high-risk behaviors in motorcyclists in order to prevent possible injuries.

## Introduction

Traffic injury is one of the biggest health problems which is neglected compared to other health issues. It needs effective preventive measures.^1^ Given the current trend, it is predicted that the number of deaths and disability caused by traffic injuries will increase more than 60% by the year 2020. It will be the third major cause of illnesses and injuries^2^ in the world. 

Motorcycle riders are one of the vulnerable groups in road crash.^3^ They confront the risk of death, the risk of injury and the risk of crash with pedestrian 8, 4 and 2 times. (respectively) more than car drivers.^4^ The probability of motorcycle accident is 9.3 times more than car accident.^5^ In Iran, more than 51 percent of trucking crash resulting in the death or hospitalization happen for motorcycle riders.^6^ Researchers believe that, given the high vulnerability of motorcycle riders,^7,8^ they have priority for research and intervention.^4^ Researches have shown that most traffic crash happen due to human incorrect behaviors.^9^ Although health education is one of the essential strategies to reform and change risky behaviors, health education experts had less contact with the prevention field of unintentional injuries.^10^ Therefore, correction factors for risky behavior remain unknown.^11^ One of the main goals of health education is to change the behavior. This will not be effective without understanding the behavioral patterns of health and safety training.^12^ Several behaviors related to traffic injuries and crash have been identified. Some of these behaviors include unauthorized overtaking, inappropriate speed, over-speeding, non-compliance with the yield sign when turning, non-compliance with traffic regulations, riding after drinking alcohol and not using helmets. ^13^ Studies have shown that it is not always possible to discover the motives behind these behaviors by observing the behavior of drivers.^14^ Social behaviors, including traffic behaviors are not merely physiological phenomena to have relatively stable and similar nature in everyone. They are relative and underlying phenomena which have different and diverse nature in different individuals and communities.^15^ Before examining how to reduce risky behaviors of drivers in the streets and roads, extensive theoretical studies are needed to reveal the mechanisms behind these behaviors.^14^ This important gap may ignore the pattern determining the risky behaviors in some motorcycle riders.^16^ The identification of motorcycle riders' risky behaviors is essential to maintain and improve the health of motorcycle riders and other community members. In the existing literature, the motorcycle riders riding behaviors have been less investigated using validated riding behavior scales especially in small-sized marginal districts of Iran. The aim of this study was to determine the riding patterns and risky riding behaviors of motorcycle riders in Bukan. Most Iranian motorcycle studies, at the time, had been conducted in mostly big cities with general Iranian culture, we selected Bukan, of a specific sub-culture, as a marginal small-sized Kurdish populated district in North-West of Iran where many people use motorcycles to do their jobs.

## Materials and Methods

This cross-sectional has been done on 340 motorcycle riders of Bukan for eight months from June 2015 to February of that year. 

**Study area**

Bukan is a marginal Kurdish residential area located in north-west of Iran. In order to make the data generalizable and to use random cluster sampling, the entire city was divided into 14 clusters based on the geographic areas covered by urban health centers. Then, 7 clusters were randomly selected out of these 14 clusters. By referring to motorcycle repair shops and motorcycle riders' home and workplace in each cluster, the data were gathered to achieve the projected sample size. 340 people (sample size) were equally divided between these seven clusters. 

**Sample size**

The sample size was calculated using Stata.v11, Sampsi according to parameters extracted from the study by Abedi et al. which was the most similar study to ours. Having the standard deviation of 22.96, the confidence level of 95% and accuracy of 3, a total of 227 people were estimated for initial sample size. According to the use of cluster sampling method, the estimated number was changed based on the design effect coefficient of 1.5. 340 people were selected for the final sample size. To collect the data, a researcher made checklist and MRBQ questionnaires were used. Based on the researcher-made checklist, basic variables such as age, marital status, level of education, the main purpose of using the motorcycle, wearing helmet, holding a valid riding license, motorcycle riding record and answering the phone while riding were measured. Motorcycle Rider Behavior Questionnaire (MRBQ) has 48 items. It was used to measure the motorcycle rider behavior. The questionnaire validity and reliability have been studied by Dr. Motevalliyan and his colleagues in Tehran in 2009. In this questionnaire, the answers have five options of "never=0", "seldom=1", "sometimes=2", "often=3" and "most of the times (nearly always) =4". This questionnaire measured the variables such as riding in the opposite direction, speeding, non-use of helmet, unauthorized load carrying and acrobatic movements. Based on Cronbach's alpha, the internal reliability was 73% to 93% for sub-groups. In this study, the behavior score obtained from the questionnaire was the dependent variable. Respectively, the inclusion and exclusion criteria for participants included 1) the interviewee must have used motorcycle at least three times monthly, 2) the minimum age of the interviewee must be 15 years old or more, 3) the interviewee should be a resident of the relevant city, 4) the interviewee must have been alert enough to complete the questionnaire, 5) the interviewee must have provided informed consent to participate in the study and 6) the interviewee have owned/held a motorcycle. Conditions of exclusion include 1) lack of informed consent to participate in the study, 2) lack of motivation to participate in the study and complete the questionnaire and 3) having no motorcycle. The collected data were entered into SPSS 18 for analysis. Frequency and frequency percentage were used for descriptive analysis of the variables. One-Way ANOVA Tests, T-Test and Pearson Correlation were used for analytical analysis. A P <0.05 was considered statistically significant. The risky behavior score, a numeric continuous scale, was considered as dependent variable in ANOVA test through which its mean was compared among the groups of the independent variable scale such as purpose of riding as a 3-group nominal scale. Pearson Correlation coefficient was calculated to investigate the association between MRBQ and other numeric normally distributed variables such as age. This study was approved by the Ethics Committee of Tabriz University of Medical Sciences. Informed consent was obtained from all the participants in this study for ethical considerations.

**Findings**

**Figure 1:**
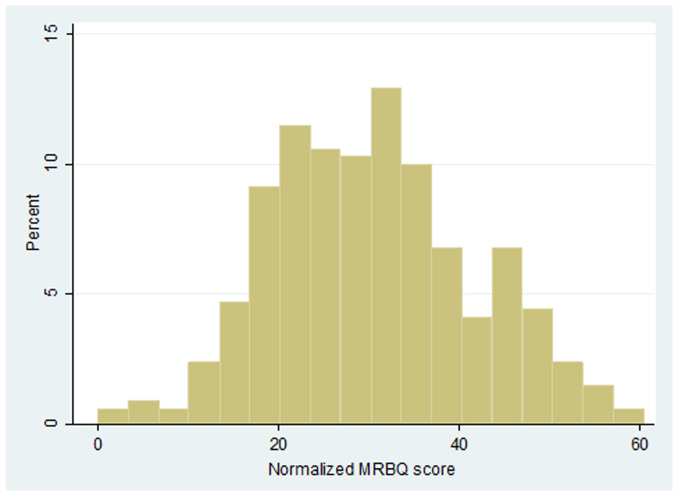
Histogram of the normalized motorcycle riding behavior questionnaire (MRBQ) score among riders in Bukan, Iran.

**Figure 2:**
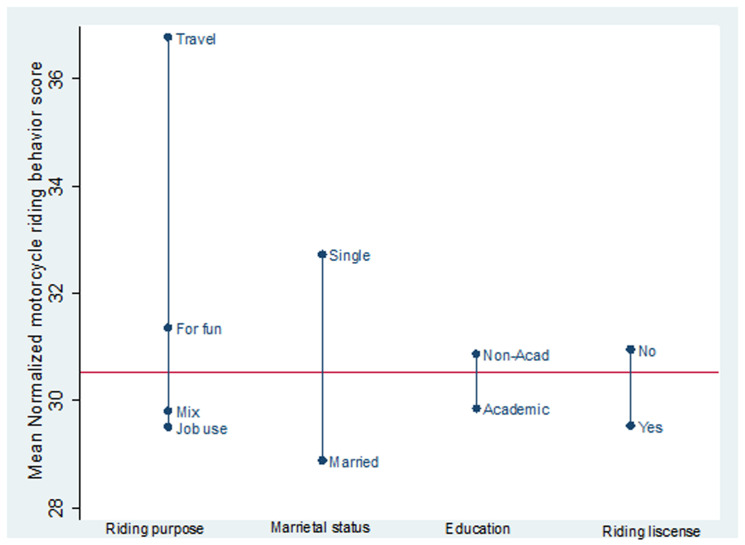
Mean normalized motorcycle riding behavior score compared for categories of various variables among riders in Bukan, Iran.

In this study, 340 motorcycle riders were investigated. All participants were male. Their mean age was 30.2 (SD=9.1, Range:17-61) years. 42.9% of study participants were single and 57.1% were married. Other demographic characteristics of participants are shown in Table 1. The most common risky behaviors possessed by at least 23% of motorcycle riders included 1) inappropriate control of motorcycle when turning, 2) taking another person without helmet by motorcycle, 3) riding without helmet, 4) taking more than one person by motorcycle, 5) exceeding the permissible speed outer city, 6) exceeding the permissible speed inside the city and 7) carrying heavy load by motorcycle. Other behaviors experienced by motorcycle riders are shown in the Table 2. Mean normalized MRBQ score was 30.5 (SD=11.2). The histogram of the MRBQ normalized score is given in Figure 1 and its mean score for various categories is presented in Figure 2.

**Table 1:** Demographic and riding-related variables of the motorcycle riders in Bukan 2015 (n=340).

Variable	Classification	Frequency(percent)
**Age**	Under 20 years	15(4.4)
****	20 to 30 years	215(63.2)
****	30 to 40 years	62(18.2)
****	40 to 50 years	35(10.2)
****	Over 50 years	13(3.8)
**Marital status**	Single	146(42.9)
****	Married	194(57.1)
**Riding record**	less than one month	3(0.8)
****	One to six months	30(8.8)
****	Six months to a year	36(10.5)
****	More than a year	271(79.7)
**Education**	Illiterate	28(8.2)
****	Primary and secondary	48(14.1)
****	High school and diploma	153(45)
****	Associate Degree	45(13.2)
****	B.A. Degree	61(17.9)
****	M.A. Degree or higher	5(1.5)
**The number of riding hours per day**	Under 2 hours	32(9.4)
****	2 to 5 hours	267(78.5)
****	5 to 7 hours	23(6.8)
****	7 to 10 hours	17(5)
****	More than 10 hours	1(0.3)
**The number of riding days per week**	Under 2 days	5(1.5)
****	2 to 4 days	106(31.2)
****	4 to 6 days	143(42.1)
****	7 days or more	86(25.3)
**Having riding license**	Has	100(29.4)
****	Doesn’t have	240(70.6)
**History of car accident**	Has	75(22.1)
****	Doesn’t have	265(77.9)

**Table 2:** Distribution of answers to questions related to the behavior of motorcycle riders.

Questions related to the behavior of motorcycle riding	Answer distribution				
How many times the following items have happened to you?	Never	Seldom	Sometimes	Often	Nearly always
When entering from the main street to a side street, you didn’t notice pedestrian who was crossing.	116	127	50	44	3
	%34	%37	%14	%12	%.9
When motorcycling, you didn’t notice pedestrian who was stepping out the parked vehicle.	54	121	131	25	9
	%15	%35	%38	%7.2	%2.9
Without realizing that a vehicle is moving on the main road or misjudging its speed, you pulled out from bystreet to the main street.	161	95	64	8	12
	%47	%27	%18	%2.4	%3.5
You ignored the “give way” sign and tried hard not to have an accident with the vehicle which has the right of way.	137	117	62	22	2
	%40.3	%34.4	%18.2	%6.5	%.6
You didn’t notice or predict that the vehicle parked in the street began pull out in front of you and you tried hard not to have an accident with your motorcycle.	42	142	109	26	21
	%12.4	%41.8	%32.1	%7.6	%6.2
On the main road, you wanted to turn right, but you might have an accident with the front vehicle due to paying too much attention to the main flow of traffic and the right side.	83	145	80	24	7
	%24.4	%42.6	%23.5	%7.1	%2.1
Due to distraction, you noticed slowing down of the front vehicle with delay and you tried hard to prevent a crash	55	114	122	27	22
	%16.2	%33.5	%35.9	%7.9	%6.5
The front vehicle showed the left-turn signal, but you tried to overtake it since you didn’t realized that.	117	92	84	41	6
	%34.4	%27.1	%24.7	%12.1	%1.8
While motorcycling, your speed is consistent with the traffic flow. At this moment, the traffic light became red and you tried hard not to have an accident.	108	124	64	34	10
	%31.8	%36.5	%18.8	%10	%2.9
You ride too close to the front vehicle so that you had trouble for stopping the motorcycle in emergency situations.	64	158	83	18	17
	%18.8	%46.5	%24.4	%5.3	%5
When turning, you turned so that you occupied the entire width of the road.	174	87	48	26	5
	%51.2	%25.6	%14.1	%7.6	%1.5
When turning, you lost the control of the motorcycle due to high speed.	122	125	67	21	5
	%35.9	%36.8	%19.7	%6.2	%1.5
In roads outside the city, you exceeded the permissible speed.	62	112	68	67	31
	%18.2	%32.9	%20	%19.7	%9.1
You ignored the speed limit at night or in early morning.	92	102	83	54	9
	%27.1	%30	%24.4	%15.9	%2.6
While motorcycling on the highway, you exceeded the permissible speed.	109	79	86	34	32
	%32.1	%23.2	%25.3	%10	%9.4
While motorcycling on the streets in residential areas, you exceeded the permissible speed.	107	97	64	44	28
	%31.5	%28.5	%18.8	%12.9	%8.2
You rejected the traffic lights to overtake the adjacent vehicle.	113	112	69	35	11
	%33.2	%33.9	%20.3	%10.3	%3.2
You rode the motorcycle with high speed on the freeway or highway.	159	76	49	38	18
	%46.8	%22.4	%14.4	%11.2	%5.3
When there was no heavy traffic, you continued motorcycling in the speed line.	96	142	64	24	14
	%28.2	%41.8	%18.8	%7.1	%4.1
You had a race with other motorcyclists.	140	71	83	37	9
	%41.2	%20.9	%24.4	%10.9	%2.6
You turned with high speed so that you scared yourself	133	113	60	26	8
	%33.1	%33.2	%17.0	%7.0	%2.4
You rode the motorcycle on one wheel (did a wheelie).	171	101	45	14	9
	%53.3	%23.7	%13.2	%4.1	%2.0
You went off road due to very quick pull away	147	128	39	21	5
	%43.2	%37.6	%11.5	%6.2	%1.5
You intentionally caused the motorcycle wheels spinning while stopping.	154	86	75	16	8
	%45.3	%25.3	%22.1	%4.7	%2.4
You unintentionally caused the motorcycle wheels spinning while stopping.	169	109	36	17	8
	%49.7	%32.1	%10.6	%5	%2.4
In darkness, you rode the motorcycle with the lights off.	172	88	43	20	17
	%50.6	%25.9	%12.6	%5.9	%5
When turning, you attempted to brake or slow down the speed.	56	75	46	80	83
	%16.5	%22.1	%13.5	%23.5	%24.4
When turning, you start gear shifting.	30	103	79	74	54
	%8.8	%30.3	%23.2	%21.8	%15.9
You lost the control of the motorcycle (for example, the steering wheel was shaken) due to high speed.	250	158	84	29	19
	%14.7	%46.5	%24.7	%8.5	%5.6
When passing over wet road, your motorcycle trembled.	72	114	122	26	6
	%21.2	%33.5	%35.9	%7.6	%1.8
A driver intentionally bothered you or put you in danger.	128	72	91	38	11
	%37.6	%21.2	%26.8	%11.2	%3.2
You carried heavy load with your motorcycle.	111	70	88	46	25
	%32.6	%20.6	%25.9	%13.5	%7.4
You carried more than one passenger with your motorcycle.	72	70	85	92	21
	%21.2	%20.6	%25	%27.1	%6.2
You had an accident with a parked vehicle but you didn’t wait for the vehicle driver.	193	66	47	25	9
	%56.8	%19.4	%13.8	%7.4	%2.6
You rode a defective motorcycle.	102	153	43	24	18
	%30	%45	%12.6	%7.1	%5.3
You rode the motorcycle without a helmet.	55	62	107	76	40
	%16.2	%18.2	%31.5	%22.4	%11.8
You carried a person without helmet by your motorcycle.	55	76	66	94	49
	%16.2	%22.4	%19.4	%27.6	%14.4
Before motorcycling, you took the medication affecting the motorcycling.	163	86	42	37	12
	%47.9	%25.3	%12.4	%10.9	%3.5
You noticed with delay the stopped vehicle opening vehicle door and you tried hard not to have an accident.	84	123	68	35	30
	%24.7	%36.2	%20	%10.3	%8.8
You crossed the intersection when the traffic light was red.	107	141	35	33	24
	%31.5	%41.5	%10.3	%9.7	%7.1
You rode the motorcycle in the opposite direction of traffic.	118	127	50	37	7
	%34.7	%37.4	%14.7	%10.9	%2.1
You rode the motorcycle on the sidewalk	102	131	69	28	10
	%30	%38.5	%20.3	%8.2	%2.9
You used mobile phone while motorcycling.	104	118	92	17	9
	%30.6	%34.7	%27.1	%5	%2.6
You rode the motorcycle without eyeglasses. (do not choose "never" if eyeglasses have not been prescribed for you)	171	74	68	21	6
	%50.3	%21.8	%20	%6.2	%1.8
You smoked while motorcycling.	170	81	57	21	11
	%50	%23.8	%16.8	%6.2	%3.2
You carried passengers with your motorcycle.	204	75	39	16	6
	%30	%22.1	%11.5	%4.7	%1.8
When using a helmet or glasses, the glass was blurred.	115	120	67	26	11
	%33.8	%35.3	%19.7	%7.6	%3.2
While motorcycling, you used helmet without the strap.	135	105	57	27	15
	%39.7	%30.9	%16.8	%7.9	%4.4

There was a significant relationship between the purpose of riding a motorcycle and risky behavior score according to the results of One-Way ANOVA test (p<0.05). There was also a significant relationship between the marital status and MRBQ score (p <<0.05). Based on Pearson correlation coefficient, there was an inverse and weak significant linear relationship between age and MRBQ score (p-value<0.05, r = -0.18). However, there seemed also to exist a nonlinear relationship between age and MRBQ score such that risky behaviors were affected by age mostly after age of 30. The multivariate linear regression analysis was developed to detect potential general predictors of risky behaviors based on MRBQ score (Table 3). The purpose of riding initially significant in multivariate model was removed after adding variables on riding experience and average daily riding. No significant association was detected between the variables of riding license status, education level, and accident history with MRBQ score in multivariate analysis. 

**Table 3:** Multivariate analysis of factors affecting the behavior score of motorcycle riders in Bukan, Iran.

Variables	Reg. coefficient	Standard Beta	Significance	95% CI
Age>=30	-2.7	-0.11	0.034	-5.1- 0.2
Lacking a riding license	2.3	0.09	0.08	-0.3- 4.8
Riding experience>1 year	-7.8	-0.28	<0.001	-10.7- 4.9
Average daily riding	Reference group: <2 hours			
2-5 hours per day	4.9	0.18	0.014	1- 8.8
>=5 hours per day	7.6	2.5	0.003	2.7- 12.5
Regression constant	29	3	<0.001	23.1- 34.9

## Discussion and Conclusion

In this study, it was observed that motorcycle riders experienced the risky behaviors including the inappropriate control of motorcycle when turning, taking pillion passenger without helmet, riding without helmet (88%), taking more than one pillion passengers by motorcycle, exceeding the permissible speed outside and inside the city, and carrying heavy load by motorcycle. In the current study, the factors such as age, marital status and purpose of using the motorcycle were significantly associated with the risky behaviors of motorcycle riders. Moreover, the amount of riding and riding experience were also independent predictors of behavior score having a higher power to exclude predictors from the multivariate model except for age and lack of riding license. Motorcycle riders' risky behaviors decreased with age. This may be due to the reduction of sensation seeking, attention attracting and high selfishness which are the causes of risky behaviors among younger motorcyclists. Single people experienced more risky behavior compared to married individuals. Low-risk behavior in the married motorcycle riders may be due to emotional connection and sense of belonging towards their family as well as higher age and riding experience. Risky driving behaviors can also be influenced by uncontrolled emotions. According to statistical reports, in most countries of the world the main cause of traffic accidents is aggressive driving. Aggressive driving behavior refers to the type of driving in which the driver drives his or her vehicle to an unsafe and uncontrolled way.^1^ Stress, violence and adventure, emotional instability, depression and insecurity are associated.^2^ It is according to the proprietary rules of motorcycle driving in Iran: motorcycle riders, including the driver and pillion passenger, should use safety helmets while riding a motorcycle. Motorbike drivers must sit on a motorcycle while driving and are not allowed to give a ride to another person unless the motorbike is fitted with a complete saddle for the pillion passenger. Among the motorcycle riders who had a variety of purposes for using the motorcycle, the type of experienced behavior was different. The most high-risk behaviors were observed when the motorcycle riders used motorcycle for travelling. This may be due to the long distance which should be passed by motorcycle rider to reach the destination. In a study (entitled "The incentive of motorcycle riders having risky behaviors for using the motorcycle") conducted by Zamani and et al. on 32 motorcycle riders in Tehran in 2008, four purposes were mentioned to use motorcycle. Depending on the purpose of motorcycle riders in using the motorcycle, there were different risky behaviors.^17^ In the current study, the motorcycle riders' risky behaviors were different depending on the purpose of motorcycle riders in using the motorcycle. The results of a study conducted by Mokhtari and et al. in Kerman in 2012 showed that more than half of motorcycle riders had experienced the risky behaviors including riding without helmet, taking more than one person by motorcycle and the fail to use helmets by the passengers.^18^ In another study conducted in Shiraz, 11.2% of participants stated that "they had always the behavior of using helmet". It is almost consistent with the rate of helmet usage in our study. In this study, half of motorcycle riders stated that they had the behavior of taking more than one person by motorcycle. The rate of helmet usage was low among the motorcycle passengers.^19^ It confirms the current study. Generally, according to a universal report, the behavior of using the helmet among Iranian motorcycle riders is reported to be 13-15%.^20^ In a study (entitled "Motorcycle riders, easy riding or easy killing") conducted by Wick in, Germany, the rate of helmet usage was 98.8%.^21^ This may be due to the lack of institutionalized culture of helmet usage and the lack of adequate law enforcement for motorcycle riders in Iran. In a study conducted by Pourhossein and et al. on 89 motorcycle riders in Sari in 2003, 15.8% of motorcycle riders have passengers on their motorcycle.^22^ It confirms the current study. In another study conducted by the sexton in Scotland in 2006, motorcycle riders had four outstanding high-risk behaviors including exceeding the permissible speed inside the city, running red lights and control errors such as the inability to control the motorcycle when turning, turning off the motorcycle when turning or facing obstacles and inability to control the motorcycle when moving at high speed^23^ These results overlap to the current study results. In another study conducted on 4183 individuals in, India to identify the high-risk behaviors of motorcycle riders, 60% of motorcycle riders stated that they experienced the malfeasances such as over speed and taking more than one person by motorcycle.^24^ In the present study, age and marital status were factors associated with lower scores on risky behaviors. This result differs from Yau findings (Hong Kong, 2004) which have shown that there is no relationship between the age of motorcycle riders with risky behavior and injury severity.^25^ This difference may be due to the different age range of motorcycle riders in these two studies. The findings of another study in New Zealand, ^26^ Spain^27^ and Taiwan^28^ have emphasized the factor of low age in motorcycle riders' risky behaviors. In a study (entitled "the investigation of the personality type of accident in injured motorcycle riders of Yazd") conducted on 304 motorcycle riders by Baghianimoghadam in 2004, the results showed that most risky and unsafe behaviors were related to young age group of 13 to 24 years. The lowest risky behavior belonged to the age group of 35 years and above.^29^ In an investigation carried out in India, it was observed that very young adolescents wanted to play the role of adults by riding the motorcycle, but they experienced risky behaviors and crash.^30-33^ In another study conducted by Elliot and colleagues, the results showed that factors including age, experience and annual distance traveled by motorcycle rider determine the risky behavior.^8^ In the current study, it was shown that travelling increases risky behavior score potentially due to the long distance which should be passed by motorcycle rider to reach the destination which in turn encourages the rider to overlook safety measures. In a case study conducted by Lardelli and colleagues in Spain from 1993 to 2003, there was a significant relationship between the low age and risky behavior of motorcycle and reducing the risk of accident.^27^ In the present study, like the studies mentioned above, there was a significant relationship between the low age and risky behavior of motorcycle. In a case study conducted by Moskal colleagues in France, the overall results showed that travel increases risky behaviors and consequently the risk of crash.^34,35^

In this study, there is also a significant association between travelling and high-risk behavior. Researches show that drivers' behavior is different in large and small towns. In big cities, drivers are more angry and confused. The authors believe that most of the motorcycle riders have the required knowledge regarding safe and unsafe behaviors. Therefore, knowledge-based interventions alone will not be adequately effective. A study conducted in South Asia has shown that educational programs alone were not effective in reducing the risk behaviors of motorcycle riders. According to the authors, the occurrence of these behaviors and ultimately the damage on motorcycle riders can be reduced by focusing on important risk factors. Contrary to current opinion, motorcycle crashes do not happen only by chance. They are preventable phenomena. The methods of controlling are different due to their different causes. The issue of road accidents is largely behavioral from the perspective of the World Health Organization and can be largely prevented by modifying individual and societal behaviors. Despite the seriousness of the issue of motorcyclists' vulnerability in Iran, little research has been conducted to suggest that these studies be based on case reports and focus on the behavior of drivers.

**Limitations**

As referring to homes and companies buying and selling the motorcycles, some motorcycle riders may not be willing to cooperate for fear of being associated with the police and judicial authorities. However, this doesn’t seem to substantially affect the results because presumably 2% to 3% of motorists in each cluster didn’t participate in the study.

**Acknowledgement**

The authors want to appreciate and gratitude friendly and sincere cooperation of Leili Abedi, Dr. Mahmoudi and others who have helped us in the course of the study. 
